# Athletes treated for inguinal-related groin pain by endoscopic totally extraperitoneal (TEP) repair: long-term benefits of a prospective cohort

**DOI:** 10.1007/s10029-023-02815-x

**Published:** 2023-06-30

**Authors:** R. R. Meuzelaar, L. Visscher, F. P. J. den Hartog, E. A. Goedhart, E. J. M. M. Verleisdonk, A. H. W. Schiphorst, J. P. J. Burgmans

**Affiliations:** 1 Department of Surgery/Hernia Clinic, Diakonessenhuis, Utrecht/Zeist, The Netherlands; 2 Department of Surgery, Erasmus University Medical Center, Rotterdam, The Netherlands; 3 Sports Medical Centre Royal Netherlands Football Association/FIFA Medical Centre of Excellence, Zeist, The Netherlands

**Keywords:** Inguinal-related groin pain, IRGP, Totally extraperitoneal procedure, TEP, Long-term follow-up

## Abstract

**Purpose:**

Inguinal-related groin pain (IRGP) in athletes is a multifactorial condition, posing a therapeutic challenge. If conservative treatment fails, totally extraperitoneal (TEP) repair is effective in pain relief. Because there are only few long-term follow-up results available, this study was designed to evaluate effectiveness of TEP repair in IRGP-patients years after the initial procedure.

**Methods:**

Patients enrolled in the original, prospective cohort study (TEP-ID-study) were subjected to two telephone questionnaires. The TEP-ID-study demonstrated favorable outcomes after TEP repair for IRGP-patients after a median follow-up of 19 months. The questionnaires in the current study assessed different aspects, including, but not limited to pain, recurrence, new groin-related symptoms and physical functioning measured by the Copenhagen Hip and Groin Outcome Score (HAGOS). The primary outcome was pain during exercise on the numeric rating scale (NRS) at very long-term follow-up.

**Results:**

Out of 32 male participants in the TEP-ID-study, 28 patients (88%) were available with a median follow-up of 83 months (range: 69–95). Seventy-five percent of athletes were pain free during exercise (*p* < 0.001). At 83 months follow-up, a median NRS of 0 was observed during exercise (IQR 0–2), which was significantly lower compared to earlier scores (*p* <0.01). Ten patients (36%) mentioned subjective recurrence of complaints, however, physical functioning improved on all HAGOS subscales (*p* <0.05).

**Conclusion:**

This study demonstrates the safety and effectivity of TEP repair in a prospective cohort of IRGP-athletes, for whom conservative treatment had failed, with a follow-up period of over 80 months.

## Introduction

Long-standing groin pain in athletes is a common condition, with an estimated prevalence of 5–10% [1]. The exact cause of this pain is often difficult to deduce and the etiology is generally multifactorial, therefore successful treatment remains challenging [2]. Different terms for long-standing groin pain in athletes are interchangeably used throughout literature, adding confusion to this already complex issue [3]. The Doha agreement has attempted to resolve this problem [4]. Accordingly, terminology is based on history and physical examination dividing athletes into categories, of which inguinal-related groin pain (IRGP) will be discussed and utilized throughout this manuscript. IRGP is defined as pain located in the inguinal canal region and tenderness during palpation of the inguinal canal [4]. Previously used terms include sportsman’s hernia, athletic pubalgia, Gilmore’s groin, sport’s hernia and inguinal disruption, but these are not preferred since no true hernia exists [3].

Multidisciplinary evaluation and treatment are highly recommended in athletes with IRGP. Treatment generally starts with conservative measures, which consist of rest, analgesics and physical therapy [5, 6]. In our high-volume hernia clinic a sports medicine physician, radiologist and hernia surgeon make up the multidisciplinary team. After diagnosis of IRGP, standard conservative treatment comprises of three months of active physiotherapy. Only if conservative treatment fails, this is followed by totally extraperitoneal (TEP) repair.

As patients that suffer from IRGP are typically young, active and healthy, some controversy exists concerning the implantation of a permanent mesh in regard to chronic pain and foreign body feeling. When positioning a heavyweight polypropylene mesh (Prolene^®^) in the preperitoneal space, a low incidence of chronic postoperative pain (CPIP) has been demonstrated in inguinal hernia surgery [7]. Furthermore, young age has not been associated with an increased risk of CPIP after endoscopic TEP repair [8]. Also, previous results showed TEP repair was effective in relieving pain in this specific patient group and offered a quick return to sport activities, with no safety related trade-offs. Roos et al. displayed that these benefits last up to a median of 19 months of follow-up, where a majority of patients were pain free and able to exercise at 100% intensity [9].

Little is known about the long-term effects of TEP repair for IRGP in athletes. It is important to know whether this treatment strategy still benefits these patients years after the initial procedure, as recurrence of complaints may occur after resumption of high-level physical activities and chronic exertion. The present study aims to determine whether TEP repair remains an effective measure in terms of pain, recurrence and re-operation, new symptoms, return to original level of sport, sport intensity and physical functioning in the very long-term for this group of patients. We expect that athletes with IRGP who undergo TEP repair will maintain positive outcomes at very long-term follow-up.

## Methods

### Patients

Patients that participated in the original, prospective cohort study (TEP-ID-study) were approached by a written letter for additional follow-up [9, 10]. The TEP-ID-study included 32 athletes with IRGP, who were diagnosed and treated by a multidisciplinary team from September 2014 through to December 2016. These athletes were professional- and amateur sportsmen, who did not benefit from conservative treatment (which consisted of refraining from sports for at least 6 weeks and 12 physiotherapy sessions), and consequently underwent TEP repair for IRGP. Patient selection was performed by an experienced sports medicine physician using the working method defined by the Manchester Consensus Conference on inguinal disruption [11]. In our high-volume hernia clinic, patients were examined by a hernia surgeon and additional imaging by means of ultrasound, pelvic X-ray and MRI was performed. The TEP-ID-study had a median follow-up of 19 months (range: 5–31 ) in 30 patients. Further details of the systematic work-up are available in the previous publication [8].

Informed consent was newly acquired since the original study protocol did not include very long-term follow-up. The present study protocol was approved by the Medical Research Ethics Committees United (MEC-U) and approval was given by the local ethics board. This manuscript adheres to the Strengthening the Reporting of Observational Studies in Epidemiology (STROBE) guidelines [12].

### Data collection

Relevant patient data were collected retrospectively from the electronic patient file to acquire baseline characteristics, consisting of age, side of operated groin, recurrence, re-operation and other relevant postoperative sequelae directly related to the TEP repair. Patients who consented to participate in the present study were subjected to a single telephone conversation, during which two questionnaires were filled out. The first questionnaire was designed specifically for this study. The questions are illustrated in Table 1. Secondly, physical functioning was assessed using the Copenhagen Hip and Groin Outcome Score (HAGOS) [13]. Data were stored in accordance to the Dutch General Data Protection Regulation (GDPR).

**Table 1:** Questionnaire assessing pain, recurrence, re-operation, new symptoms, sport intensity and return to sport

	Questions	Answers
1	Do you experience pain during exercise?	NRS 0–10 (0 = no pain, 10 = worst pain possible)
2	Have your initial complaints returned? (recurrence)	Yes/ no
3	Did you have a second operation of the affected groin? (re-operation)	Yes/ no
4	Do you experience new symptoms in the operated groin?	Yes/ no
5	What is the intensity of sport you practice now compared to the intensity of sport you played before surgery of your groin?	0–100%
6	How many days did it take to return to your original level of sport after surgery of your groin?	Time in days (−1 = never returned to original level of sport)

### Outcomes

The primary outcome of the present study was pain during exercise, measured on the Numeric Rating Scale (NRS). This score was compared to the patients’ pain scores before treatment, 3- and 19 months after treatment, which were extracted from the TEP-ID-study. Additionally, scores were classified into four NRS score strata (no pain 0; mild pain 1–3; moderate pain 4–7; severe pain 8–10).

Secondary outcomes were recurrence of initial complaints, re-operation, occurrence of new symptoms, the intensity of sport, time to return to the original level of sport and physical functioning. Recurrence of initial complaints was defined as patient-reported recurrence of inguinal-related groin pain or discomfort of the operated groin. Re-operation was considered any type of inguinal hernia surgery on the ipsilateral side of the original procedure due to recurrence of complaints or new symptoms. New symptoms were defined as any new pain or discomfort in the operated groin area, as reported by patients. The intensity of sport currently practiced was compared to the preoperative situation and expressed as a percentage. Time to return to the original level of sport was the amount of days it took to fully recover after TEP repair and achieve their preoperative level of sport. Physical functioning was determined using the HAGOS. This is a patient-reported questionnaire, in which six different domains are addressed: pain, symptoms, activities of daily living (ADL), sport/ recreation, participation, and quality of life (QoL). These domains are scored from 0 to 100, where 0 is the worst possible score and 100 is the best possible score in that specific domain. No additional outpatient appointments were made and no physical examination was performed.

### Statistical analysis

Statistical analyses were performed with SPSS, version 26 (IBM Corp., Armonk, NY, USA). Demographics and baseline characteristics are reported descriptively. Descriptive statistics include median and interquartile range (IQR) or mean and standard deviation (SD) for continuous variables, and frequency counts with percentages for categorical variables. Relevant differences between pre- and postoperative parameters are presented as median with IQR and were analyzed with the Wilcoxon signed rank test for continuous data. For discrete data, McNemar’s test (binary) and the Wilcoxon signed rank test (nominal/ordinal) were used. For between-group comparisons the Mann–Whitney U test was utilized. Patients lost to follow-up were excluded from the final analyses. The same applies for missing data, due to pairwise analyses.

## Results

**Fig. 1:**
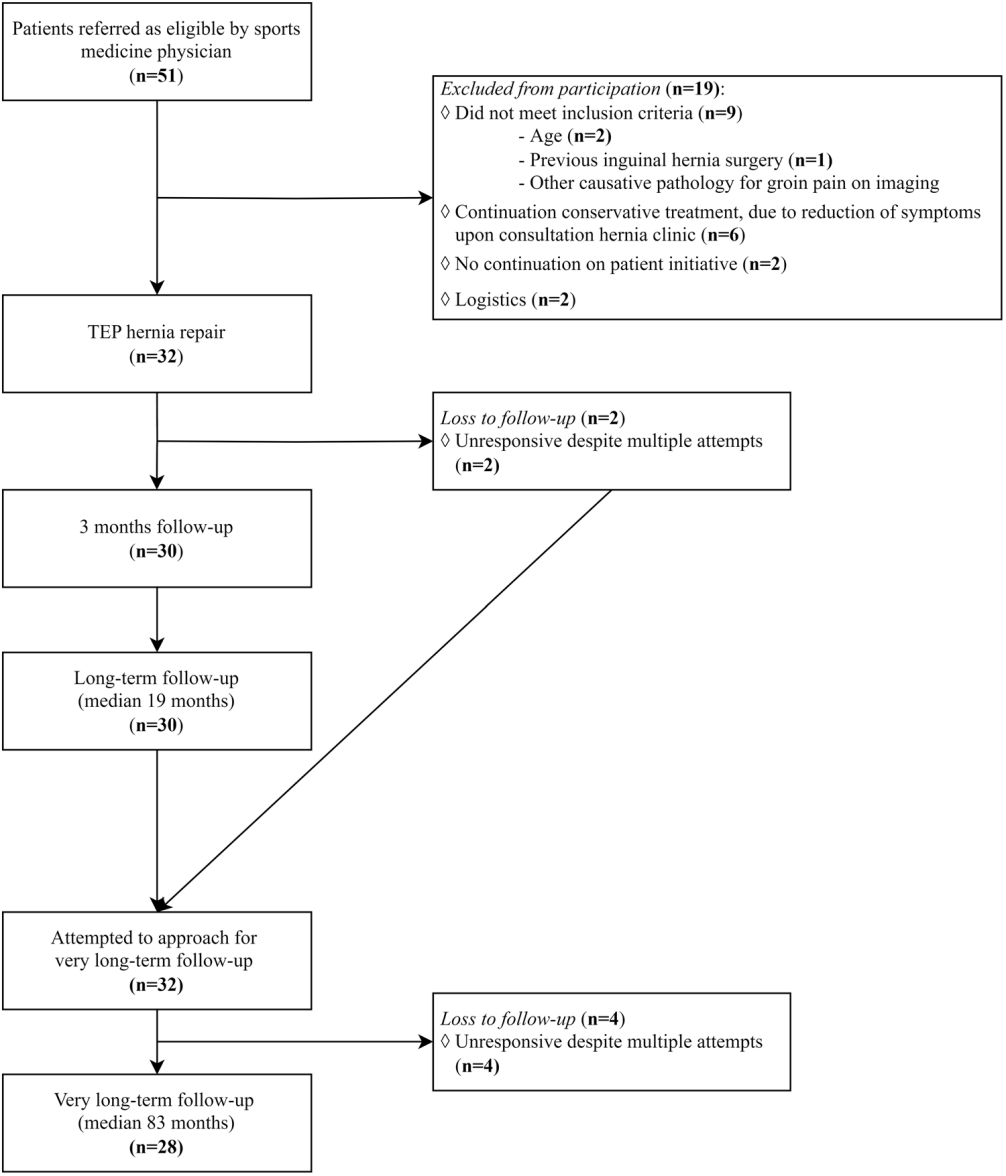
Flowchart of inclusions and follow-up. *TEP *totally extraperitoneal

From October 11th 2022 until December 12th 2022, patients received a phone call answering two questionnaires. Out of 32 male participants in the TEP-ID-study, 28 patients (88%) were available for follow-up with a median age of 30 years (IQR 27–43). Four patients remained unresponsive despite multiple attempts to get in contact by phone or e-mail (Figure. 1). Median follow-up time was 83 months (range: 69–95). Table 2 summarizes the baseline characteristics and the answers of the first questionnaire (Table 1).

**Table 2:** Baseline characteristics (*n* = 28). Q refers to the corresponding questions depicted in Table 1

Age in years, median (IQR)	30 (27–43)
Side of operated groin, *n* (%)	
Right	13 (46)
Left	13 (46)
Bilateral	2 (8)
Q1. Pain score during exercise (NRS), median (IQR)	0 (0–2)
Q2. Recurrence of complaints, *n* (%)	10 (36)
Q3. Re-operation, *n* (%)	0 (0)
Q4. Newly reported symptoms, *n* (%)	6 (21)
Q5. Intensity of sport (%), median (IQR)	80 (43–80)
Q6. Time in days until original level of sport was achieved after surgery, median (IQR)	90 (44–180)

### Pain

The median preoperative pain score during exercise was 8 (IQR 7–8) and this decreased to 0 (IQR 0–2) at 83 months follow-up (*p* < 0.001). A significant NRS score reduction was also observed between 3- and 83 months after treatment (*p* = 0.014). However, no significant difference was found between 19- and 83 months follow-up (*p* = 0.718).

**Fig. 2:**
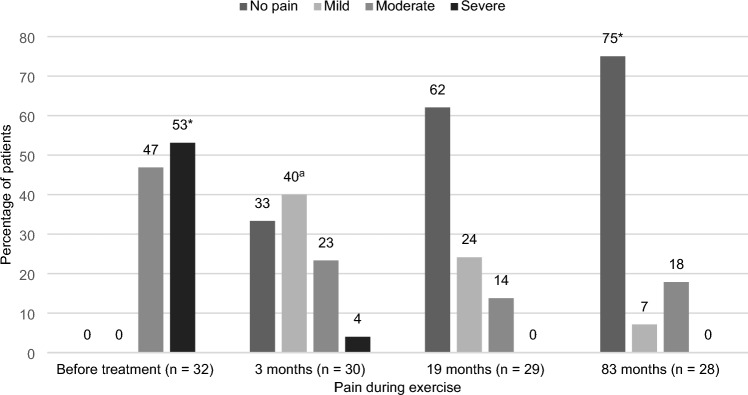
Pain during exercise at the following timepoints: before treatment, 3-, 19- and 83 months after treatment. Pain scores (NRS) were stratified as follows: no pain 0; mild pain 1 – 3; moderate pain 4 – 7; severe pain 8 – 10. *Before treatment – 83 months *p *< 0.001, ^a^3 months – 83 months *p =* 0.021

Seventy-five percent of the patients were pain free (*p* < 0.001) and none reported severe pain (NRS 8–10) during exercise. Ten patients (36%) mentioned subjective recurrence of initial complaints. However, when observing the outcomes of their HAGOS, these recurrences were not perceived as painful or limitative. Six patients described new symptoms and no patients received additional surgical therapy. Mild pain (NRS 1–3) significantly reduced (*p* = 0.021) between 3- and 83 months postoperatively. Similarly, severe pain reduced significantly at 83 months follow-up (*p* < 0.001) compared to the preoperative situation. For all other categories, comparing scores between 83 months follow-up and earlier timepoints, no significant differences were objectified (Fig. 2).

A significant correlation was observed between recurrence and NRS score (*p* = 0.002), which was higher in the group whom reported recurrence of complaints. For patients experiencing new, different symptoms there was no relationship with NRS score.

### Resumption of sports

The median time to return to their original level of sport was 90 days (IQR 44–180). Three patients (11%) did not return to their original level of sport. The median intensity of sport was 80% (IQR 43–80) and 46% of the athletes were able to exercise at 100% intensity.

### Physical functioning

**Fig. 3:**
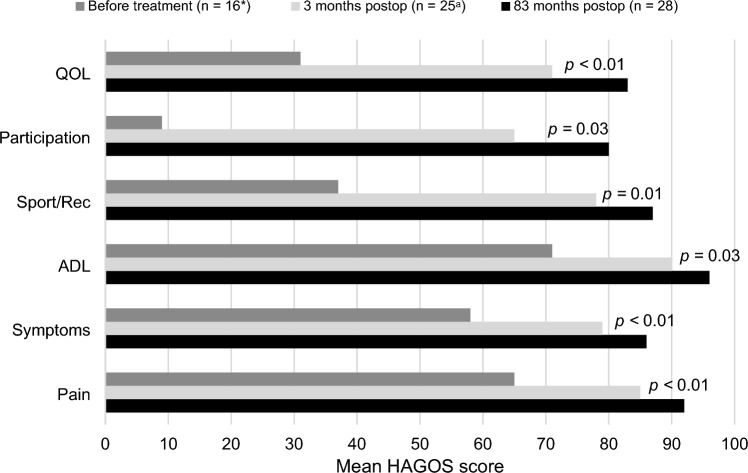
Mean HAGOS scores representing physical functioning at different timepoints. *ADL *activities of daily living, *Rec* recreation, *QOL* quality of life. The P-values are for comparisons between 3- and 83 months after treatment. *Except for pain and sport/rec *n*=15. ^a^Except for pain and symptoms *n*=24 and sport/rec *n*=23

Improvement on all subscales of the HAGOS was shown at 83 months postoperatively, with high scores on all domains (>80.0) The highest score was observed for ADL (96.3) and the lowest for participation (80.4), which is similar to the TEP-ID-study (Fig. 3). Every subscale displayed a significant increase compared to scores before treatment and 3 months after treatment (*p* < 0.05).

## Discussion

This prospective cohort study demonstrates the effectiveness of TEP repair at 83 months follow-up for the majority of athletes suffering from IRGP who did not respond to conservative treatment. To our knowledge, this study has the longest follow-up for TEP repair in this selected patient group so far.

The diagnosis and treatment of long-standing IRGP in athletes remain challenging considering the condition is not well-defined in research as well as in clinical practice and different pathology often coexists [14]. There is no consensus about the exact underlying pathogenesis and different hypotheses are reported in the literature. The most cited theory is weakness of the posterior abdominal wall of the inguinal canal with or without dilation of the inguinal ring.[15–17]. To add more confusion, the clinical relevance of diagnostic imaging remains unclear. Symptomatic patients may exhibit normal findings on imaging modalities and vice versa. Nevertheless, MRI and ultrasound can be useful to exclude other (co)existing abnormalities [4, 14, 18]. A multidisciplinary approach, including physiotherapists, sports medicine physicians, radiologists and surgeons is necessary to adequately select patients that may benefit from a surgical intervention.

Many different treatment modalities have been proposed, but only few prove to be of high quality [19]. Conservative management consists of passive physical and/or exercise therapy or steroid injections. Surgical treatment options include open or laparoscopic repair or adductor tenotomy. In some cases, inguinal hernia repair is combined with adductor tenotomy or additional neurotomy [19–21]. Physical examination and patient history give a reliable indication of which patients will benefit from surgical treatment. Moderate evidence exists for TEP repair being superior to nonsurgical treatment [19]. In a randomized controlled trial (RCT) athletes treated surgically had lower pain scores and higher rates of returning to sport activities 12 months postoperatively [22]. Limited evidence is available for all other treatments options for IRGP [19].

In accordance with the Doha agreement, we selected patients based on the criteria described for IRGP [4]. An RCT using a similar systematic work-up to our study, showed that TEP repair was less painful than open repair in the first month. At 12 months follow-up, all patients of both groups (*n* = 65) were pain free and the two treatment options were assumed to be equally effective [23]. These excellent outcomes could be attributed to the selection procedure used. The follow-up period of more than 6 months should be appropriate to assess CPIP [24]. One major limitation compared to our study is that they did not incorporate patient-reported outcome measures (PROMs) questionnaires. Using only one parameter to represent physical functioning, namely pain during sport activities, oversimplifies this complicated matter. Brans et al. did include the HAGOS questionnaire and presented significant improvement of all the subscales after TEP repair, which is in line with our results. They clearly described clinical signs as defined by the Manchester Consensus Conference [11]. Sadly, the reported follow-up period of 6 weeks is too short to draw any conclusions regarding CPIP.

Many other studies report non-specific diagnostic criteria. Also, there is a lack of uniformity in reporting these criteria. Paajanen et al. included patients based on patient history, physical examination and imaging studies in a multidisciplinary manner [22]. On the contrary, patients experiencing tenderness over the pubic symphysis and adductor tendons were also regarded eligible. Notably, in this RCT six patients received open tenotomy in addition to TEP repair and in all patients a mesh was placed bilaterally, even if complaints were unilateral. Bilateral mesh placement was supported by the theory that this would prevent later operation on the non-affected side due to induced preperitoneal scarring. It is debatable whether this is justified, considering mesh-related complaints, e.g. foreign body reaction, which may lead to excessive scarring and consecutive functional consequences in a formerly asymptomatic groin.[25]. Kluin et al. considered similar criteria to diagnose athletes and 85% were pain free after a 12-month follow-up period. Yet, there was inconsistency in the surgical technique used. In some cases a transabdominal preperitoneal (TAPP) approach was used or this was converted into TEP [26]. Van Veen et al. noted that 91% of patients could return to sport activities after 3 months. However, all patients with groin pain (*n* = 55) were included, this definition lacks precision. For retrospective studies it is difficult to clearly specify diagnostic criteria since data are collected retrospectively. Edelman and Le et al. used perioperative characteristics and treatment codes to identify eligible patients [20, 27].

Studies with longer follow-up are of low quality, mainly due to their design. Srinivasan et al. prospectively reported no recurrence of symptoms at 46 months follow-up, yet diagnosis was executed poorly and the sample size was small (*n* = 15) [28]. In our cohort, ten (36%) patients self-reported recurrence of complaints, but physical functioning improved on all subscales which implies they experienced little to no pain or limitations. Another prospective cohort study (*n* = 28) recorded a pain reduction of 100% at 3 months postoperatively (mean follow-up: 15 months) [29]. Although they have used some form of a multidisciplinary approach, inclusion criteria and outcomes were not well-defined. Paajanen et al. used pain during palpation of the pubic tubercle to identify eligible patients and excluded other causes of groin pain clinically and radiologically. At four years, the vast majority of athletes benefitted from surgery (*n* = 41) and mesh was again placed bilaterally, with 5% of patients receiving additional tenotomy. In addition, there was no clear definition of the primary outcome or record of their telephone questionnaire assessing the latter. Even though all these studies applied different selection protocols, TEP repair was repeatedly proven to be effective for athletes with IRGP. A possible explanation for these positive outcomes is the significant correlation Serner et al. reported between higher treatment success and lower quality studies [19].

Comparing the results of the present study to NRS scores at 19 months follow-up from the TEP-ID-study in this patient cohort, no significant difference was present. This would imply that the favorable outcome does not change over time and TEP repair remains effective in this specific population [9]. We believe a probable reason for this is found in the etiology of IRGP. Weakness of the posterior wall is reinforced by mesh placement which leads to pain relief [30]. Sport intensity decreased to a median of 80% (IQR 43–80) in comparison to the median maximum intensity of 100% at 19 months postoperatively (IQR 90–100). It is reasonable to assume the intensity of sport decreased over the years due to patients becoming older and that the decrease is not attributable to recurrence of complaints.

The lack of a control group poses a substantial weakness of this and the previous study. However, it would have been ethically debatable to perform an RCT on this uncommon condition with a watchful waiting group, since these patients were urgently in need of pain relief. Another limitation of our study is the possibility of recall bias concerning the questionnaires. When asking the number of days it took to return to their original level of sport after surgery, which happened around seven years ago, it is understandable this is merely an estimation of the exact number of days. Furthermore, follow-up was conducted solely by telephone, so there was no chance to perform physical examination. It could have been interesting to ask patients with recurrent complaints or new symptoms to visit the outpatient clinic and objectively assess these.

Finally, this study demonstrates the safety and effectivity of TEP repair in a prospective cohort of athletes with IRGP, for whom conservative treatment had failed, with a follow-up period of over 80 months. We emphasize the importance of a multidisciplinary, systematic work-up for adequate patient selection to gain high success rates of surgical treatment in this specific patient group and to enable generalizability of the current results. To decrease heterogeneity and aid interpreting results between studies, there is a need for more high quality research conducted in larger cohorts with the use of the definitions and criteria of the Doha agreement.
